#  

**DOI:** 10.1111/cas.15589

**Published:** 2022-12-22

In an article^1^ titled “Exosomal zinc transporter ZIP4 promotes cancer growth and is a novel diagnostic biomarker for pancreatic cancer” by Haoyi Jin, Peng Liu, Yunhao Wu, Xiangli Meng, Mengwei Wu, Jiahong Han, Xiaodong Tan, the following errors were published:

All the transmission electron microscope images (Fig 1C, Fig 3B, Fig 6E) are uploaded in error and these are corrected in the figures below.

Figure 1
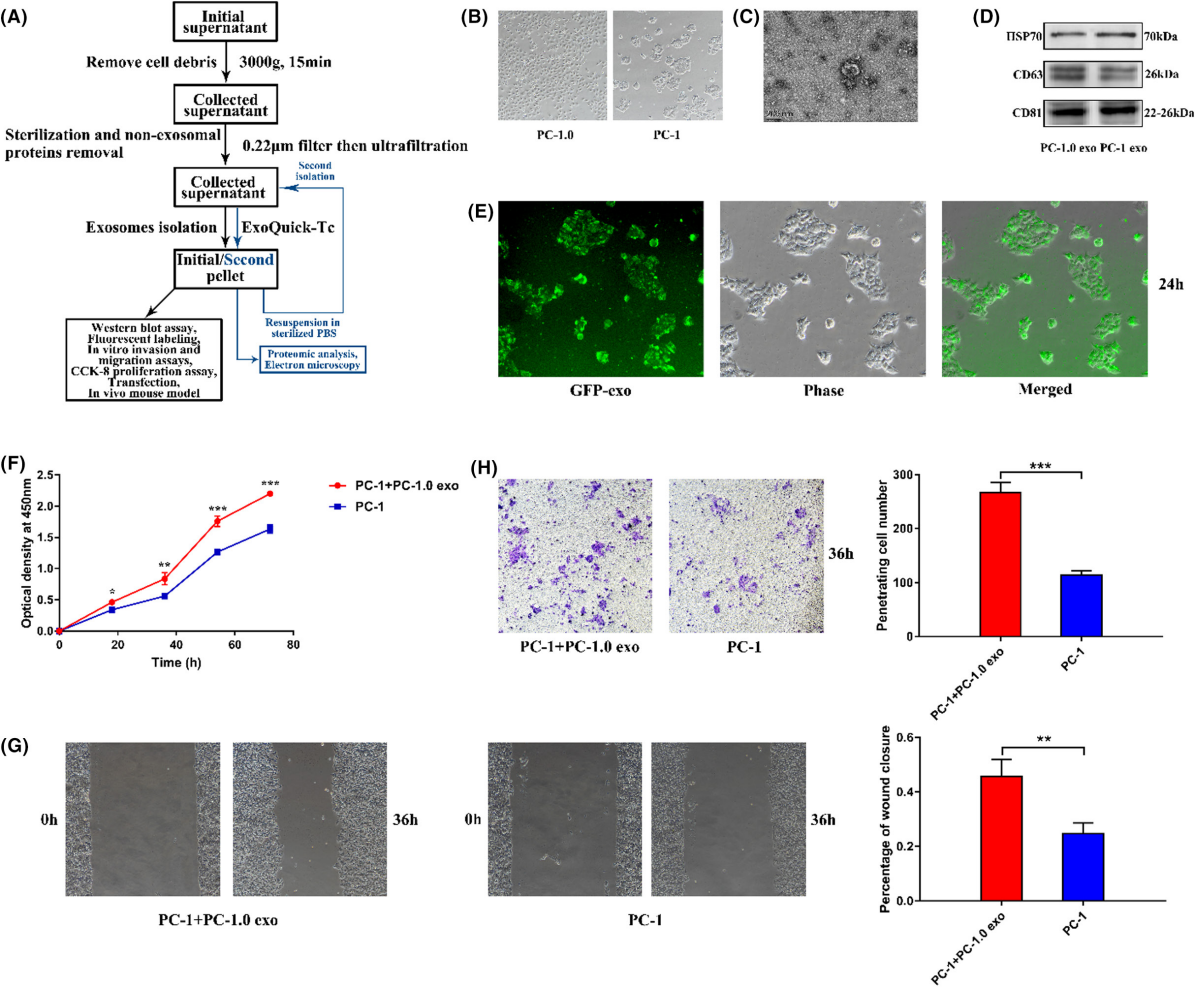


Figure 3
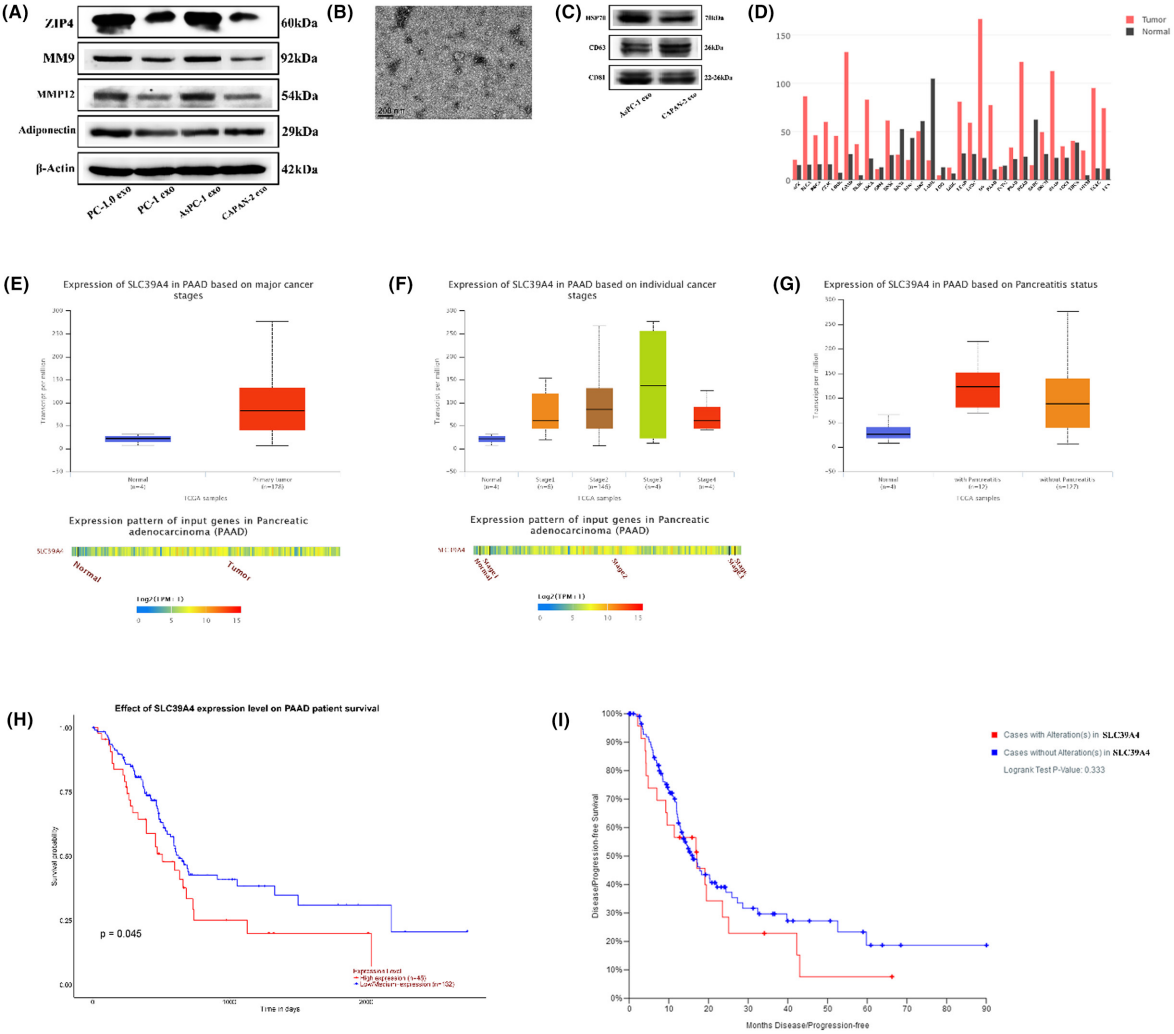


Figure 6
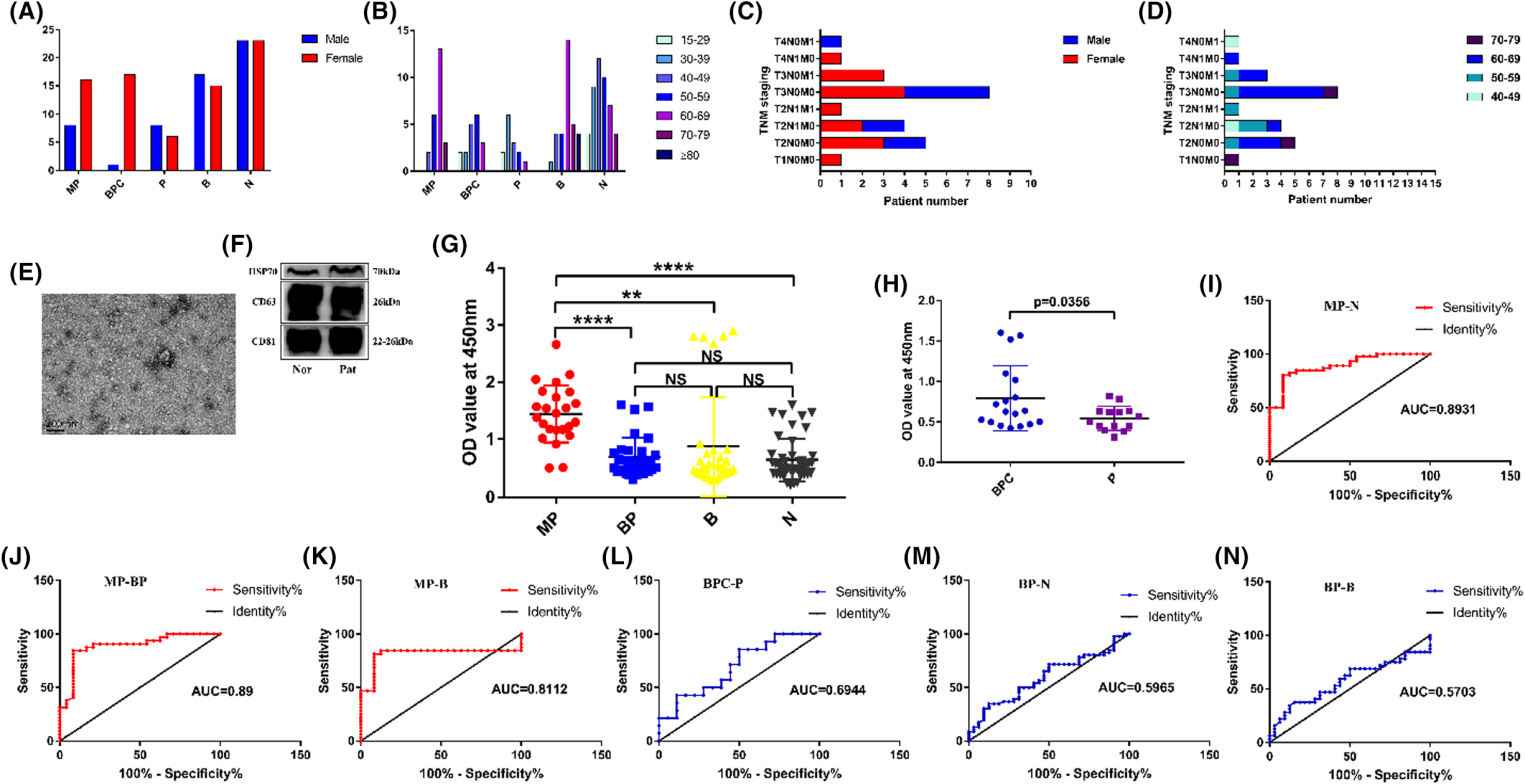


The authors apologize for the error.

## References

[1] Jin H , Liu P , Wu Y , et al. Exosomalzinc transporter ZIP4 promotes cancer growth and is a noveldiagnostic biomarker for pancreatic cancer. Cancer Sci. 2018;109:2946‐2956. doi:10.1111/cas.13737 30007115 PMC6125444

